# Machine learning in the detection of dental cyst, tumor, and abscess lesions

**DOI:** 10.1186/s12903-023-03571-1

**Published:** 2023-11-06

**Authors:** Vyshiali Sivaram Kumar, Pradeep R. Kumar, Pradeep Kumar Yadalam, Raghavendra Vamsi Anegundi, Deepti Shrivastava, Ahmed Ata Alfurhud, Ibrahem T. Almaktoom, Sultan Abdulkareem Ali Alftaikhah, Ahmed Hamoud L Alsharari, Kumar Chandan Srivastava

**Affiliations:** 1 Department of Public Health Dentistry, Saveetha Dental College, Saveetha Institute of Medical and Technical Sciences, Saveetha University, Chennai, Tamil Nadu India; 2 Department of Periodontics, Saveetha Dental College, Saveetha Institute of Medical and Technical Sciences, Saveetha University, Chennai, Tamil Nadu India; 3 Department Department of Preventive Dentistry, College of Dentistry, Jouf University, 72345 Sakaka, Saudi Arabia; 4 Oral Surgery Department, Institute of Dentistry, Queen Mary University of London, London, E1 2AD UK; 5 Present Address: College of Dentistry, Jouf University, 72345 Sakaka, Saudi Arabia; 6 Department of Oral & Maxillofacial Surgery & Diagnostic Sciences, College of Dentistry, Jouf University, 72345 Sakaka, Saudi Arabia; 7 Department of Oral Medicine and Radiology, Saveetha Dental College, Saveetha Institute of Medical and Technical Sciences, Saveetha University, Chennai, Tamil Nadu 602105 India

**Keywords:** Dental Images, Artificial Intelligence, Digital Image Processing, GLRLM, GLCM

## Abstract

**Background and Objective:**

Dental panoramic radiographs are utilized in computer-aided image analysis, which detects abnormal tissue masses by analyzing the produced image capacity to recognize patterns of intensity fluctuations. This is done to reduce the need for invasive biopsies for arriving to a diagnosis. The aim of the current study was to examine and compare the accuracy of several texture analysis techniques, such as Grey Level Run Length Matrix (GLRLM), Grey Level Co-occurrence Matrix (GLCM), and wavelet analysis in recognizing dental cyst, tumor, and abscess lesions.

**Materials & Methods:**

The current retrospective study retrieved a total of 172 dental panoramic radiographs with lesion including dental cysts, tumors, or abscess. Radiographs that failed to meet technical criteria for diagnostic quality (such as significant overlap of teeth, a diffuse image, or distortion) were excluded from the sample. The methodology adopted in the study comprised of five stages. At first, the radiographs are improved, and the area of interest was segmented manually. A variety of feature extraction techniques, such GLCM, GLRLM, and the wavelet analysis were used to gather information from the area of interest. Later, the lesions were classified as a cyst, tumor, abscess, or using a support vector machine (SVM) classifier. Eventually, the data was transferred into a Microsoft Excel spreadsheet and statistical package for social sciences (SPSS) (version 21) was used to conduct the statistical analysis. Initially descriptive statistics were computed. For inferential analysis, statistical significance was determined by a *p* value < 0.05. The sensitivity, specificity, and accuracy were used to find the significant difference between assessed and actual diagnosis.

**Results:**

The findings demonstrate that 98% accuracy was achieved using GLCM, 91% accuracy using Wavelet analysis & 95% accuracy using GLRLM in distinguishing between dental cyst, tumor, and abscess lesions. The area under curve (AUC) number indicates that GLCM achieves a high degree of accuracy. The results achieved excellent accuracy (98%) using GLCM.

**Conclusion:**

The GLCM features can be used for further research. After improving the performance and training, it can support routine histological diagnosis and can assist the clinicians in arriving at accurate and spontaneous treatment plans.

## Introduction

The most traditional way to confirm a dental cyst, tumor, or abscess lesion is by performing an incisional or excisional biopsy [1]. A dental practitioner or a specialist may carry this out this invasive procedure. The sample is then sent to an oral pathologist who examines the tissue under a microscope to check for abnormality. The success of this method depends on how accurately the needle is placed, skill and experience of the oral pathologist [2, 3]. An unsuitable, unrepresentative sample is unproductive, and the patient might have to undergo repeated procedures. A second, unnecessary biopsy may be required if the patient receives inadequate medical care at any stage. It is often time-consuming and is subjected to unacceptable inter and even intra-observer variations [4, 5]. There are various other ways to determine the presence or extent of oral cancer. Radiographs can be especially helpful in assessing the presence and extent of oral cancer as they provide a non-invasive and comprehensive view of the affected areas, aiding in early detection and precise treatment planning. One of the most valuable, standard, and routinely used diagnostic tools are radiographs, which are an adjunct to clinical examination in diagnosing dental diseases [6, 7]. It is a painless and quick method that is the most preferred and reliable [8].

Effective diagnosis and early intervention are crucial in preventing dental complications, with various radiographic modalities playing a key role in this process. Dental abscesses and cysts are common periapical conditions that can affect the maxillary and mandibular jaws [9], and their timely detection and treatment can save patients from considerable discomfort and expense. Clinical classifications help differentiate between these lesions, each with its own distinct causes [10, 11]. Detecting and treating dental issues before they progress to more significant complications may save time, money, and pain. Various dental radiographic modalities such intraoral periapical, panoramic, and cone beam computed tomographic radio-graphs plays a pivotal role in diagnosis and treatment planning [12–15]. Oral panoramic images play an important role is identification of lesions which related to not only to the oral diseases but also to various systemic disorders. This eventually assist the clinician to plan, treat and make referral for systemic conditions [16, 17]. By analyzing the resulting radiographic images with distinct patterns of intensity fluctuations, computer-aided image analysis may be able to identify aberrant tissue masses. Automatic segmentation will simplify lesion analysis, making it easier to spot and identify lesions in the clinic. Oral panoramic pictures cannot reliably distinguish between lesions due to their uniformly smooth, spherical or oval borders [18, 19]. To help pathologists in the detection of cysts and tumors, researchers have devised quantitative methodologies for computer-aided diagnosis utilizing dental panoramic radiographs [20, 21].

Oral cancer segmentation isolates oral cancer in a medical image. This can be done manually or automatically with computer vision. Automatic segmentation is more accurate but computationally expensive than manual segmentation, which is time-consuming and imprecise [22–24]. The process begins with the segmentation of the lesion. Rough sets, fuzzy logic & neural networks are examples of soft computation methods that may be used to arrive at solutions that are more intelligent, affordable, and understandable than those reached by more conventional means [25]. By efficiently analyzing and predicting ill-nesses based on data stored in the database, artificial neural networks provide a useful tool for clinicians. Implementing neural networks on a Field Programmable Gate Array (FPGA) enables more adaptability in software-defined hardware [26]. FPGAs are ideal for real-time applications because of their compact size and fast processing rates [27, 28].

GLCM (Gray Level Co-occurrence Matrix) is a statistical approach that analyses the spatial relationship between image pixels. It builds a matrix showing the frequency of each grey level combination at a given distance and direction. GLRLM (Gray Level Run Length Matrix) is a statistical method that measures the length of consecutive pixels with the same grey level in a picture. It builds a matrix of run length frequencies for each grey level. GPGA (Gray Level Peak Grouping Analysis) is a statistical method that counts and sizes peaks in an image's grey level histogram. It builds a matrix of peak size frequencies for each grey level. Wavelet analysis is a mathematical method that breaks down a signal into wavelets. Each signal wavelet has a particular frequency and scale. Analyzing wavelet coefficients can extract characteristics from photos.

Abnormalities were detected from non-enhanced oral panoramic radiographs using Wavelet Image processing. Image characteristics that could identify tumors, abscesses, and cystic lesions were analyzed using texture analysis. Features of the image were derived from the GLCM, comprising correlation, contrast, homogeneity, & energy [29]. When a wavelet transform was used on the pictures, the coefficients were calculated [30]. The GLCM & wavelet transform image characteristics were used as input vectors for the neural network training process.

Images were sorted into "normal" and "abnormal" categories based on the network's conclusions. Segmentation based on in-tensity was used to locate the lesion area in the irregular pictures. For more precise diagnosis of oral tumor, cyst, or abscess, an artificial neural network [31]was created on FPGA with the help of a system generator & Xilinx ISE 14.3. The objective of this study was to compare the diagnostic accuracy in detecting dental cysts, abscesses as well as tumor lesions via image processing on panoramic dental images to the actual diagnosis.

## Materials and Methods

### Ethics

Before starting the study, ethical clearance was obtained from the institutional ethics committee of Saveetha University (IHEC/SDC/FACULTY/22/PERIO/104).

### Study characteristics

The purpose of the study was to detect dental cysts, abscesses as well as tumor lesions via image processing based on panoramic dental radiographs. The Department of public health dentistry designed an in vitro, retrospective study with the help from Department of Oral Medicine and Radiology, Saveetha dental college, and the Hospital.

Null Hypothesis (H0): There is no significant difference in the accuracy of texture analysis techniques, including Grey Level Run Length Matrix (GLRLM), Grey Level Co-occurrence Matrix (GLCM), and wavelet analysis, in recognizing dental cyst, tumor, and abscess lesions.

Alternate Hypothesis (Ha): There is a significant difference in the accuracy of texture analysis techniques, including Grey Level Run Length Matrix (GLRLM), Grey Level Co-occurrence Matrix (GLCM), and wavelet analysis, in recognizing dental cyst, tumor, and abscess lesions.

### Sample characteristics

Digital panoramic radiographs with cystic, tumor or abscess lesions were retrieved. Radiographs of poor diagnostic quality (significant overlap of teeth, diffused image, or distortion) were excluded. The sample size was determined based on the re-search conducted by Ingrid et al.; 2003, N = 172 (calculated using G-power) (at 95% power and 5% α-error) [32].

### Study protocol

A total of 172 digital panoramic radiographs were selected based on the pre-determined inclusion and exclusion criteria. Tumor lesions in the selected orthopantomograph (OPGs) include ameloblastoma, odontoma, ameloblastic fibroma, adenomatoid odontogenic tumour, hemangioma, enostosis, exostosis, cementoblastoma, torus mandibularis, torus palatinus, myxoma, osteoma, and osteoid osteoma. In addition to above-mentioned tumor lesion, dental cyst and abscess lesions were also selected. These measurements were taken with a digital orthopantomograph (PlanmecaProOne; Helsinki, FINLAND) at the Department of Oral Medicine and Radiology, Saveetha Dental College and Hospital. Patient’s identification data including age, gender, and the date of radiograph date were all recorded for each patient.

**Fig. 1:**
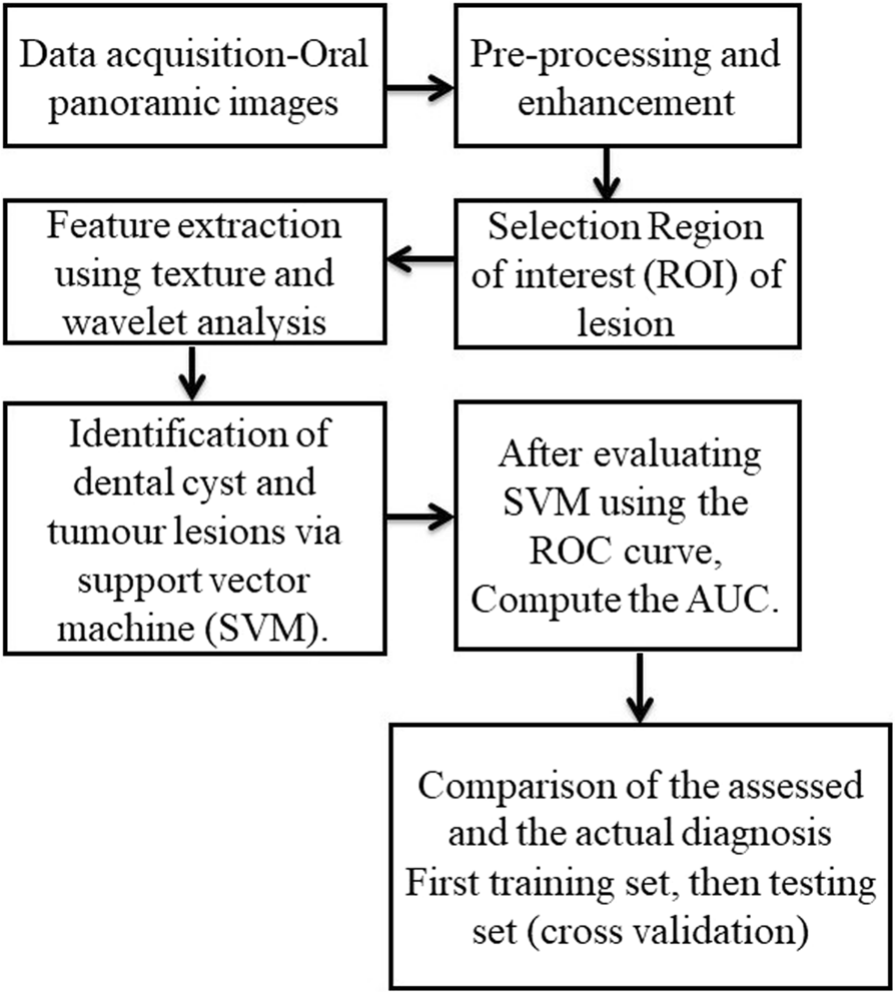
**A** decomposition at level 1 **B** decomposition at level3

In the present study, the characteristics of dental panoramic radiographs were used to create methods for differentiating cyst and tumor lesions. The correlation between the evaluated diagnosis and the actual diagnosis followed (Fig. 1).

**Fig. 2:**
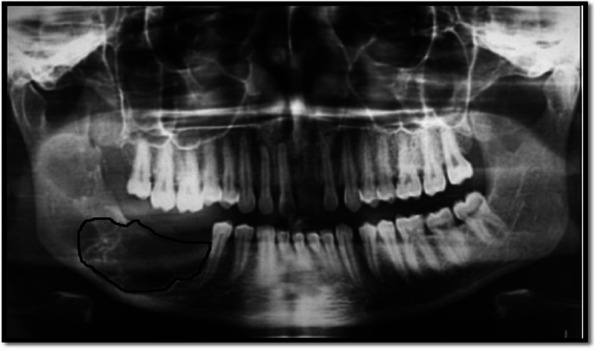
Schematic representation of methodology

**Fig. 3:**
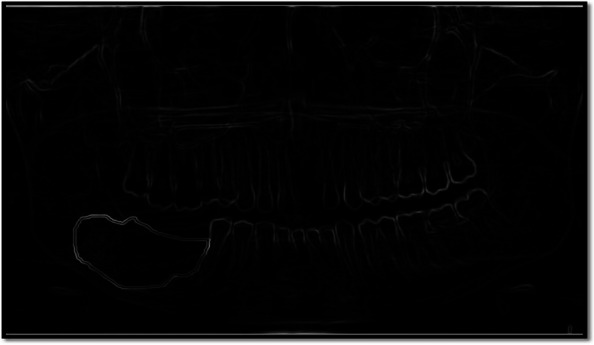
Data Acquisition-Oral panoramic image of ameloblastoma (dental tumor)—Data Acquisition-Oral panoramic image of ameloblastoma (dental tumor). Increased radiolucency with ill-defined margins is observed in the jaw's lower right posterior tooth region, with related tooth structures missing

**Fig. 4:**
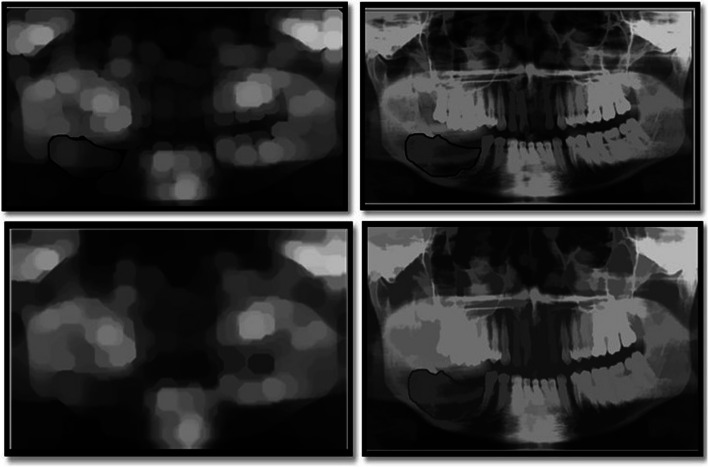
Preprocessing and enhancement of the obtained OPG image—It shows the Preprocessing and enhancement of the obtained OPG image. In this step, the removal of artifacts and noise from the Image is carried out

**Fig. 5:**
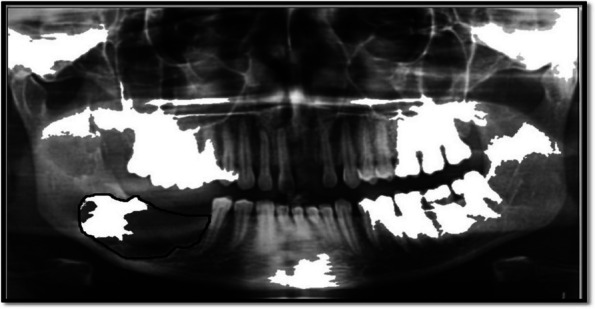
Morphological processing for extracting Region of Interest – It shows the Morphological processing for extracting the Region of Interest. In this step, the lesion is associated with the nearby anatomical landmark and is separated from the OPG image

**Fig. 6:**
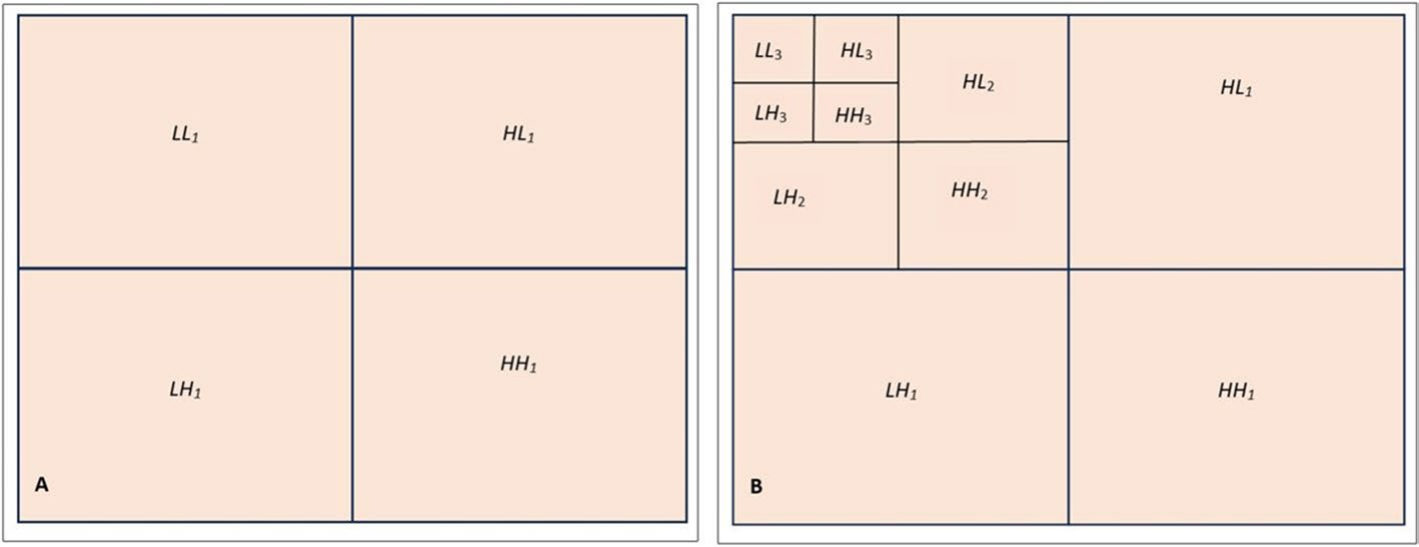
It shows the regional maxima superimposed on the original for enhancing the Region of Interest (ROI). Here, the lesion is segmented and undergoes wavelet and texture analysis (GLCM, GLRLM)

A. *Data acquisition:* A set of dental panoramic images were collected from Saveetha Dental College and Hospital. Ground truth image was considered as a reference (Fig. 2).
B. *Preprocessing and Enhancement:* Pre-processing is required to enhance the picture quality of dental panoramic radiographs since they are difficult to interpret. This step facilitates subsequent stages of feature extraction that are easier and more trustworthy. The radiographs were cleaned up to remove any artefacts or background noise during the preprocessing procedure. Noise was reduced and smoothed down using the gaussian filter, allowing for better contrast in dental panoramic radiographs. Noise was reduced by using a 3-by-3-square median filter. In order to get rid of the outliners without reducing the image's sharpness, a median filter is used since it is less sensitive to extreme values. When the median filter is applied, abnormalities become sharper in the homogenous background. The area around the lesion has to be amplified. The purpose of Imadjust is to enhance images. The intensity of a grayscale picture is modified by imadjust to a new value that is 1% of the da-ta at the extremes of brightness. This improves the image's contrast (Fig. 3).
C. *Selection Region of interest (ROI) of a lesion:* Dental panoramic radiographs with cyst and tumor lesions served as the regions of interest for this study. A trained operator manually identified all lesions in the radiographs, and these findings were then verified by a radiologist. With the mass placed in the middle of the window, a 40 × 40-pixel region of interest was extracted. The masses were then split in two: the training group and the evaluation group. The training set featured 40% cyst pictures and 40% tumor images, whereas the testing set contained 20% cyst images and 20% tumor images using one-three-hold-out cross-validation (Fig. 4).
D. *Feature extraction using texture and wavelet analysis* (Fig. 5)*:*
  - Feature extraction using texture analysis: The Image's texture is the local variations in the image's intensity. Using a co-occurrence matrix, statistical texture analysis is possible. This matrix is produced by estimating the pairwise statistics of image pixel intensity. The Grey level Co-occurrence Matrix [GLCM] was developed based on the premise that identical gray-level configurations are repeated in a texture. The co-occurrence matrix is denoted by P (i, j | d,), in which i and j are grey level readings separated by a distance d and an angle. With GLCM, it is possible to extract second-order statistical features. The GLCM includes the same number of rows and columns (Fig. 6). The following notations are used in GLCM.

μ- Mean value of P

μx- Mean value of Px

μy- Mean value of Py

σx- Standard deviation of Px

σy- standard deviation of Py

G- Size of the co-occurrence matrix

The characteristics of GLCM that were utilized in this study included energy, correlation, contrast, and homogeneity.

i. Energy: It provides the uniformity of an image. The energy of a constant image is 1.

$$Energy=\;\sum\limits_{i=0}^{G-1}\sum\limits_{j=0}^{G-1}\;P\left(i,j\right)^2$$

ii. Correlation: measures level dependence among pixels at the specified relative position to one another. For an image with multiple linear structures, correlation will be high.

$$Correlation=\sum\limits_{i=0}^{G-1}\sum\limits_{j=0}^{G-1}\frac{\left\{i,\;j\right\}\;\times P\left(i,\;j\right)\;-\;\left(u_x\;\times\;\mu_x\right)\;}{\sigma_x\;\times\;\sigma_y}$$

iii. Contrast: refers to the total amount of local differences present in an image. This indicates the degree to which p(i,j) from the diagonal element i = j influences the degree of contrast. The greater the image variation, the more diagonal P(i, j) values will be concatenated, resulting in a higher contrast.

$$contrast=\sum_{n=0}^{G-1}\;n^2\left\{\sum_{i=0}^{G-1}\sum_{j=0}^{G-1}\;P\left(i,\;j\right)\right\},\;\left|i-j\right|\;=n$$

Homogeneity: It measures the nearness of the components in GLCM with the diagonal of the GLCM matrix and is used to evaluate the homogeneity of the GLCM. Homogeneity is 1 for the diagonal elements. The Co-occurrence matrix of a heterogeneous images provide an even distribution of P (i, j) values, whereas homogeneous images include a combination of high as well as low P (i, j) values.

**Fig. 7:**
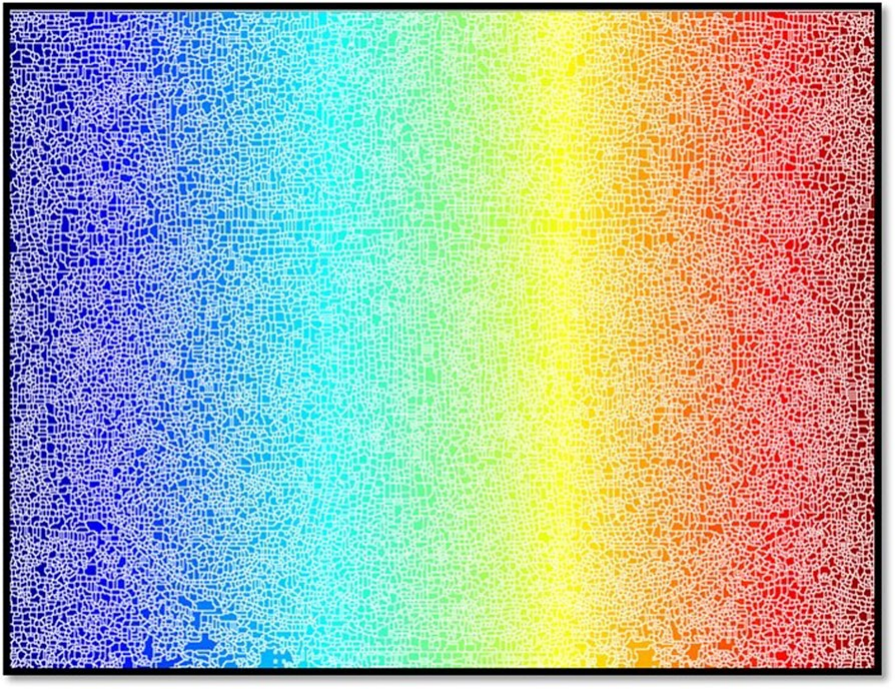
shows the Wavelet analysis for features extraction from wavelet coefficients. After this, the Image is subjected to a support vector machine classifier (SVM)

An Input vector to the neural network and the wavelet transform coefficients were given.

z. *Feature extraction utilizing Wavelet Analysis*: To approximate a picture, we can utilize matrix A with the pixel intensities at each grey level P(i,j) as its elements [10]. A is a square with dimensions of 2n × 2n, where n is A integer. Here is how the wavelet transform works: The rows of A are processed using filters G and H. The resultant matrices are HrA and LrA of size 2n2n-1. Next, the HrA and LrA column matrix is processed once again with the G and H filters, yielding four matrices HcLrA, HcHrA, LcLrA, and LcHrA of size 2n-1 × 2n-1. The LcLrA matrix is an average matrix, whereas the other matrices are more detailed matrices. It's also possible to execute many degrees of decomposition (Fig. 7).

$$Homogenity=\sum_{i=0}^{G-1}\sum_{j=0}^{G-1}\;\frac{P\left(i,\;j\right)}{1\;+\;\left|i-j\right|}$$

E. Identifying dental cysts or tumor lesions using the support vector machine (SVM) method:

Following feature extraction and selection, these data were fed into a classifier that categorize each picture to one of two classes: cysts or tumors. In order to classify these lesions, we used Support Vector Machine [SVM] technique.

The ROC curve was used in this study because of its capacity to provide a comprehensive and objective assessment. The ROC curve is the scatter plot of True Positive Fraction (TPF) as the function of False Positive Fraction (FPF). The area that is under the receiver operating characteristic curve (AUC) can be used as a criterion [33, 34].

F. Evaluate SVM using the ROC curve and compute the AUC:

The ROC curve was used in this study since it is both comprehensive and objective in its assessment [32]. The ROC curve is the scatter plot of True Positive Fraction (TPF) as the function of False Positive Fraction (FPF). The area that is under the receiver operating characteristic curve (AUC) can be used as a criterion [33, 34].

### Statistical analysis

The gathered data was transferred into a Microsoft Excel spreadsheet and SPSS (version 21) was used to conduct the statistical analysis. Initially descriptive statistics were computed. For inferential analysis, statistical significance was determined by a *p*-value of 0.05.

### Measures of performance evaluation

The effectiveness of the system is evaluated using a variety of criteria. Classification Accuracy (AC) and Mathews Correlation Coefficient (MCC) are the measures used. The Confusion Matrix is used for calculating these values. The results of a classification system's actual classifications and predictions are summarized in a confusion matrix (Kohavi and Provost, 1998) [35]. The matrix information is often used to assess the effectiveness of such systems. The confusion matrix for a two-class classifier is shown in the tabular column below.

**Table:** 

TP (True Positive) – Correct prediction of abnormal
TN (True Negative) – Correct Prediction as normal
FP (False Positive) – Incorrect prediction of abnormal
FN (False Negative) – Incorrect prediction of normal

From the confusion matrix, accuracy (AC) can be obtained:

$$\mathrm{Accuracy}\frac{(\mathrm{TP}+\mathrm{TN})}{(\mathrm{TP}+\mathrm{FP}+\mathrm{TN}+\mathrm{FN})}$$

The Matthews correlation coefficient (MCC): In machine learning, the Matthews coefficient of correlation (MCC) measures the accuracy of two-class(binary) classifications. The MCC, which yields a value between 1 and + 1, is essentially a correlation coefficient that exists between observed as well as predicted binary classifications. A coefficient of + 1 indicates a perfect prediction, coefficient 0 is no better than a random prediction, and a coefficient of -1 denotes complete disagreement between the prediction and the observation. The MCC is estimated by using:

$$\mathrm{MCC}=\frac{\mathrm{TP}\;\times\;\mathrm{TN}\;-\;\mathrm{FP}\;\times\mathrm{FN}\;}{\surd\left(\mathrm{TP}\;+\;\mathrm{FP}\right)\;\left(\mathrm{TP}+\mathrm{FN}\right)\;\left(\mathrm{TN}+\mathrm{FP}\right)\;\left(\mathrm{TN}+\mathrm{FN}\right)}$$

Clinical tests are evaluated based on their sensitivity and specificity.

- Sensitivity of a clinical test is defined as the ability of the test to identify the percentage of patients with disease, Calculated as: TP / (TP + FN)
- Specificity is defined as the ability of test to appropriately identify healthy individuals, Calculated as: TN / (TN + FP)
- Positive Predictive Value (PPV) or precision rate is the proportion of positive test results that are the true positives (accurate diagnosis). The PPV is denoted by the formula, PPV = TP / (TP + FP)

## Results

**Fig. 8:**
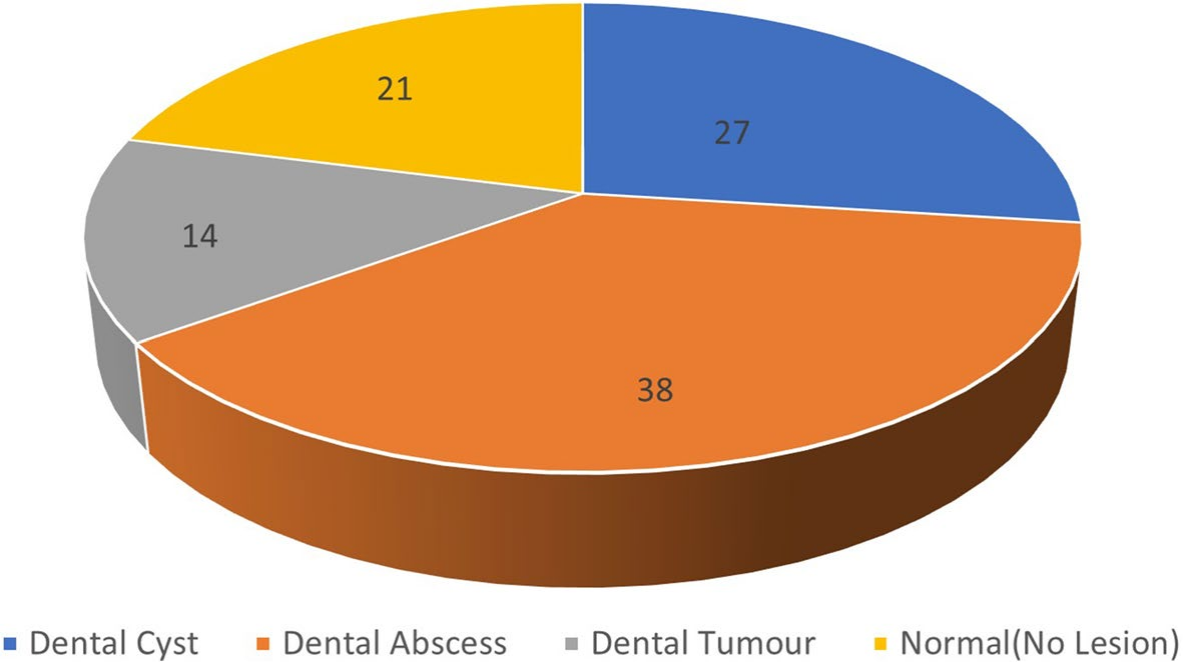
Distribution of OPG images based on dental cysts, tumors, and abscess lesions

Figure 8 shows the distribution of 172 OPG images in which 73 (38%) were dental abscesses, 52 (27%) were dental cysts, 27 (14%) were dental tumors, and 40 (21%) images were normal without any lesions.

Table 1 depicts the performance of GLCM by identifying the true negative (39), false negative (1), false positive (0), and true positive (40) by comparing the actual and predicted classifications done by confusion matrix for diagnosing dental cyst, tumor, and abscess lesions using 80 OPG images in which 40 images had the lesion and 40 images were without any lesion.

**Table 1:** Confusion matrix for GLCM features

		Predicted	
		Negative(-)	Positive( +)
Actual	Negative(-)	39(TN)	1(FN)
	Positive( +)	0(FP)	40(TP)

*TN* True Negative, *FN* False Negative; *FP* False Positive; *TP* True Positive; *GLCM *Grey Level Co-occurrence Matrix

Table 2 depicts the performance of GLCM by identifying the true negative (40), false negative (2), false positive (2), and true positive (36) by comparing the actual and predicted classifications done by confusion matrix for diagnosing dental cyst, tumor, and abscess lesions using 80 OPG images in which 40 images had the lesion and 40 images were without any lesion.

**Table 2:** Confusion matrix for GLRLM features

		Predicted	
		Negative(-)	Positive( +)
Actual	Negative(-)	40(TN)	2(FN)
	Positive( +)	2(FP)	36(TP)

*TN* True Negative, *FN* False Negative; *FP* False Positive; *TP* True Positive; *GLRLM* Grey Level Run Length Matrix

Table 3 depicts the performance of GLCM by identifying the true negative (30), false negative (4), false positive (3), and true positive (43) by comparing the actual and predicted classifications done by confusion matrix for diagnosing dental cyst, tumor, and abscess lesions using 80 OPG images in which 40 images had the lesion and 40 images were without any lesion.

**Table 3:** Confusion matrix for wavelet analysis

		Predicted	
		Negative(-)	Positive( +)
Actual	Negative(-)	30(TN)	4(FN)
	Positive( +)	3(FP)	43(TP)

*TN* True Negative, *FN* False Negative; *FP* False Positive; *TP* True Positive

Table 4 depicts the performance of GLCM, GLRLM, and wavelet analysis by identifying the true positive, true negative, false positive, and false negative by comparing the actual and predicted classifications done by confusion matrix for diagnosing dental cyst, tumor, and abscess lesions using 80 OPG images in which 40 images had the lesion and 40 images were without any lesion.

**Table 4:** Matrix for all three techniques

Value	Wavelet analysis	GLCM	GLRLM
TP	43	40	36
FP	3	0	2
FN	4	1	2
TN	30	39	40

*TN* True Negative, *FN* False Negative; *FP* False Positive; *TP* True Positive; *GLRLM *Grey Level Run Length Matrix; *GLCM* Grey Level Co-occurrence Matrix

**Fig. 9:**
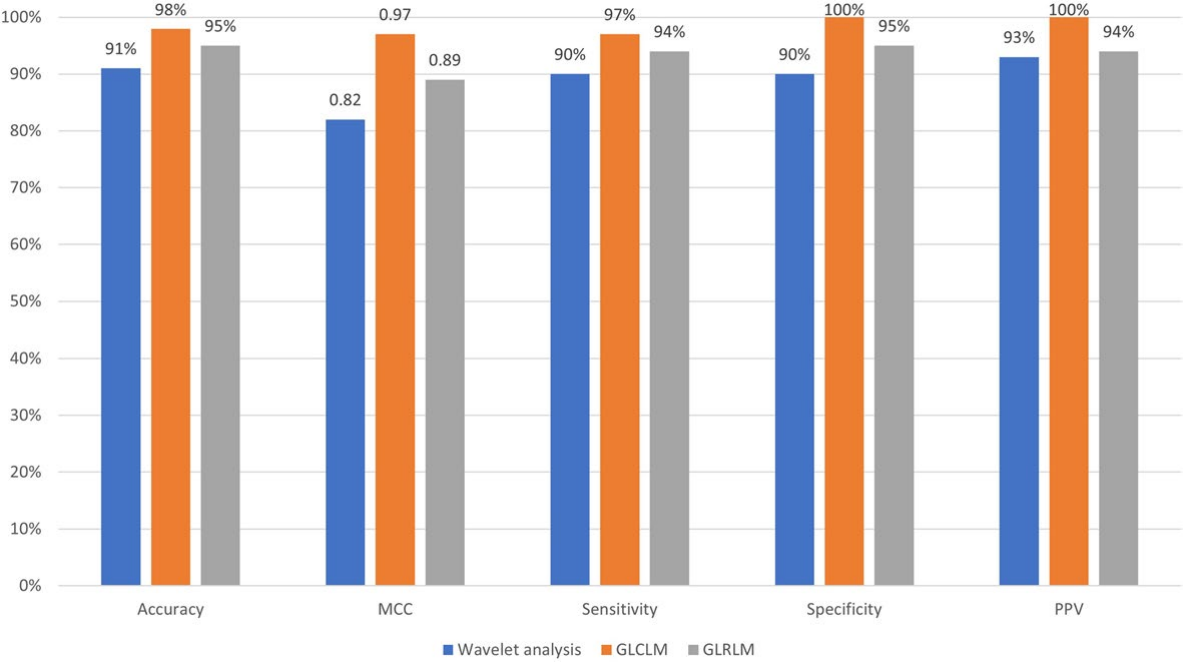
Analysis of the Performance of wavelet analysis, GLCM, and GLRLM

Table 5 demonstrates the calculated accuracy, specificity, sensitivity, Mathews Correlation Coefficient, and Precision Rate calculated using values from Table 4. Various models are used to extract texture features, and the results are inconsistent (Fig. 9).

**Table 5:** Evaluation results

Measure	Wavelet analysis	GLCLM	GLRLM
AC	91%	98%	95%
MCC	0.82	0.97	0.89
SN	90%	97%	94%
SP	90%	100%	95%
PPV	93%	100%	94%

*TN* True Negative, *FN* False Negative; *FP* False Positive; *TP* True Positive; *GLRLM *Grey Level Run Length Matrix; *GLCM* Grey Level Co-occurrence Matrix

**Fig. 10:**
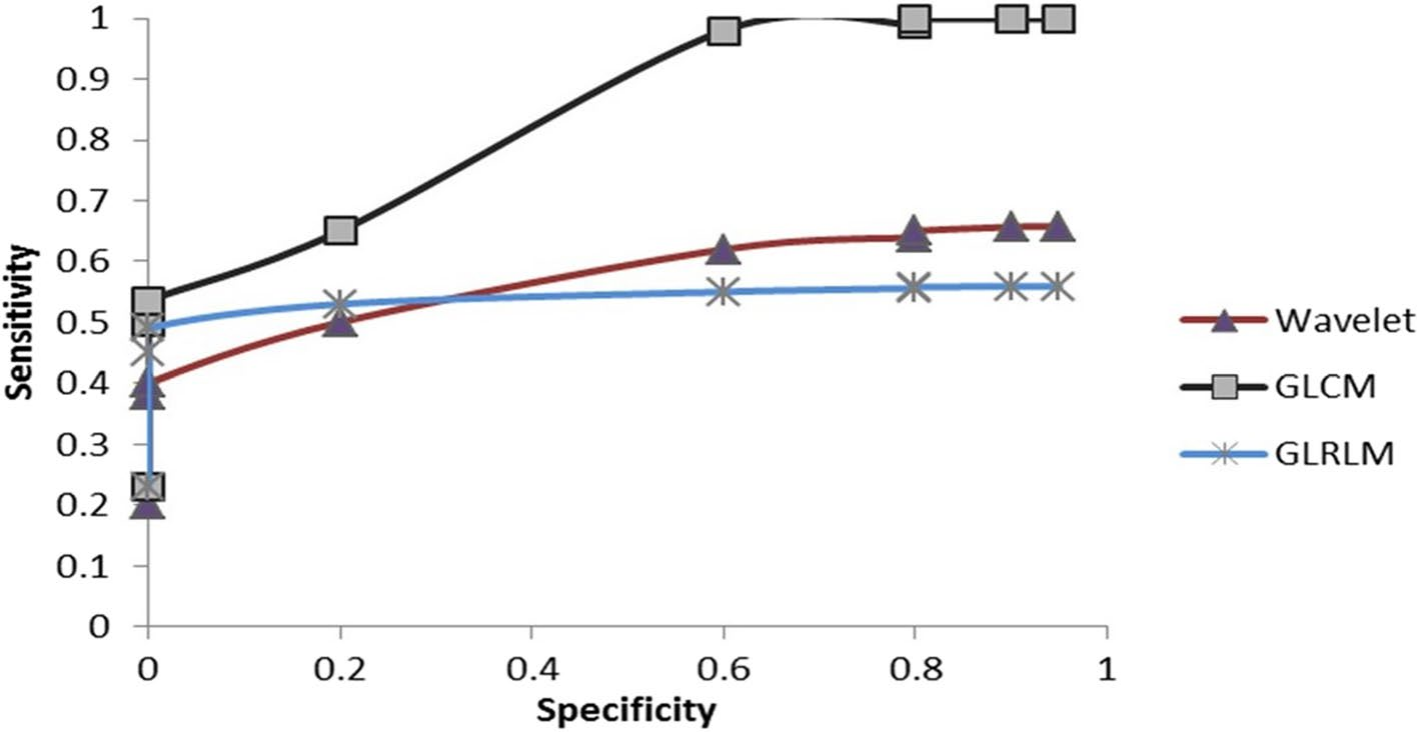
Receiver operator characteristic curve

Figure 10 shows the receiver operator characteristic (ROC) curve evaluating the performance of GLCM, GLRLM, and wavelet analysis by plotting sensitivity along the y-axis and Specificity along the x-axis. The area under the ROC curve (AUC) is a criterion.

## Discussion

Based on their morphology and protein expression, histology and immunohistochemistry currently diagnose dental tumors and cysts. A biopsy is a painful procedure that cannot be recommended every time [36]. Therefore, the computer-aided diagnosis of a dental cyst, tumor, and abscess lesions using oral panoramic images is attempted in this study [37]. This will avoid exposure to unnecessary biopsies. An intelligent, low-cost, and interpretable solution may be achieved via the use of soft computing methods such as texture analysis and neural networks for extracting the features than traditional techniques [38].

Today texture-based medical imaging has a vast number of research being done on the early detection of stroke, Automatic Diagnostic Systems for CT Liver and CT (computed tomography) images are widely utilized to diagnose liver disease and develop a computer-assisted classification system for detecting cancer using digital mammograms [39–45].

This study approaches the problem by running texture (GLCM and GLRM) and wavelet analysis to extract the features in the Image. We calibrated values by feeding the recognition and differentiation of dental cysts, abscesses, and tumour lesions using the abovementioned methods. All the methods analysed the change in the expression of each lesion. Later sensitivity, Specificity, accuracy, MMC, and PPV of the classification were calculated. All the experiments were performed using MatlabVer 7.1 on a PC Intel Pentium Centrino with 1 GB of RAM. A total of 53 dental abscesses, 17 tumors, and 52 dental cyst lesions measuring 40 × 40 pixels were transformed into wavelet analysis, GLCM, and GLRLM. The minimum and maximum values of all three lesions are different. We acquired up to 91% accuracy with wavelet analysis, 98% accuracy with GLCM, and 95% accuracy with GLRLM; utilizing the ROC curve along with computing the AUC, we acquired up to 0.9444 AUC. with FO, we estimated the AUC for GLCM up to 0.9361 and for GLRLM up to 0.8722.

The feature values from both classes overlap, but all the three lesions have different minimum and maximum values. Due to the overlapping value, this conclusion suggests that the categorization process is not straightforward (non-linear). The non-linear classification is made feasible by the maximum and minimum feature values within each class. Lesion types such as cysts have median values between 13.7650 and 213.2888, whereas tumors have mean values between 85.0038 and 252.3044. This indicates that SVM may be used to differentiate cyst lesions, abscesses, and tumors.

The texture traits were shown to be useful in identifying cysts and tumor lesions in this research. When attempting to locate a region of interest (ROI) in an image, texture is an important factor to consider. Wavelet analysis was used to extract 5 texture features, 13 texture features were extracted based on GLCM, and 7 texture features were extracted based on GLRLM. Wavelet analysis provides a set of five features: central tendency, dispersion, kurtosis, skewness, and dispersion. GLCM utilized the following measures: autocorrelation, contrast, correlation, cluster prominence and cluster shade, energy, dissimilarity, Homogeneity, information measure, maximum probability, inverse difference, inverse difference normalized, and inverse difference moment normalized. GLRLM utilized the following measures: Low Gray Level Run Emphasis, High Gray Level Run Emphasis, Short Runs Emphasis, Long Runs Emphasis, Run per-centage, Gray Level Non-uniformity, Run Length Non-uniformity.

The ROC curve was used in this study because of its capacity to provide a com-prehensive and fair evaluation ability [43]. A ROC curve is the plotting of true positive fraction (TPF) as the function of false positive fraction (FPF) [46, 47]. The area under the ROC curve (AUC) can be used as a criterion.

A strength of this method is that it is simple, non-invasive, and quick. This method holds significant promise for cancer patients by potentially enabling more personalized and targeted treatment approaches based on the specific genetic signatures identified, ultimately enhancing the effectiveness of cancer therapies. The primary objective of the research was to optimize the classification and prediction models for these cancers, which led to the identification of 96 genes that are extremely relevant to these malignancies. Nonetheless, we anticipate that the above list can be extended by employing more detailed arrays and larger training sample sets.

Although we achieved a high degree of specificity and sensitivity for diagnostic classification, we expect that the sensitivity of these models can be improved for diagnostic purposes in clinical practice with larger arrays and more samples. Research into the staging and biological behavior of malignancies, with the goal of predicting prognosis and guiding treatment, is a potential future use of these technologies. Furthermore, future research ought to be performed to improve the precision of GLRLM as well as wavelet analysis texture characteristics. Since we believe that this approach provides a robust and alternative means for identifying dental tumours, cysts, and abscesses, and given that the results indicate enhanced system performance with a larger set of training images, we recommend this method as a valuable tool in clinical practice.

## Conclusions

The accuracy of the results achieved through GLCM analysis yielded an impressive 98 percent. These GLCM features demonstrate strong potential for further exploration. Consequently, there is a pressing need for future investigations aimed at refining the precision of GLRLM and wavelet analysis texture characteristics. This supports the prospective use of these techniques as an adjunct to standard histological diagnosis. This research will aid dentists in establishing accurate diagnoses and prompt treatment plans for the benefit of their patients.

## Data Availability

The data will be available on reasonable request from the corresponding author.
